# *Camino Verde* (The Green Way): evidence-based community mobilisation for dengue control in Nicaragua and Mexico: feasibility study and study protocol for a randomised controlled trial

**DOI:** 10.1186/s12889-017-4289-5

**Published:** 2017-05-30

**Authors:** Neil Andersson, Jorge Arostegui, Elizabeth Nava-Aguilera, Eva Harris, Robert J Ledogar

**Affiliations:** 1 Centro de Investigación de Enfermedades Tropicales (CIET), Universidad Autónoma de Guerrero, Acapulco, Mexico; 2 Department of Family Medicine, McGill University, Montreal, Canada; 3 CIET, Managua, Nicaragua; 4 Division of Infectious Diseases and Vaccinology, School of Public Health, University of California, Berkeley, CA USA; 5 CIET international, New York, NY USA

## Abstract

**Background:**

Since the *Aedes aegypti* mosquitoes that transmit dengue virus can breed in clean water, WHO-endorsed vector control strategies place sachets of organophosphate pesticide, temephos (Abate), in household water storage containers. These and other pesticide-dependent approaches have failed to curb the spread of dengue and multiple dengue virus serotypes continue to spread throughout tropical and subtropical regions worldwide. A feasibility study in Managua, Nicaragua, generated instruments, intervention protocols, training schedules and impact assessment tools for a cluster randomised controlled trial of community-based approaches to vector control comprising an alternative strategy for dengue prevention and control in Nicaragua and Mexico.

**Methods/Design:**

The Camino Verde (Green Way) is a pragmatic parallel group trial of pesticide-free dengue vector control, adding effectiveness to the standard government dengue control. A random sample from the most recent census in three coastal regions of Guerrero state in Mexico will generate 90 study clusters and the equivalent sampling frame in Managua, Nicaragua will generate 60 clusters, making a total of 150 clusters each of 137–140 households. After a baseline study, computer-driven randomisation will allocate to intervention one half of the sites, stratified by country, evidence of recent dengue virus infection in children aged 3–9 years and, in Nicaragua, level of community organisation. Following a common evidence-based education protocol, each cluster will develop and implement its own collective interventions including house-to-house visits, school-based programmes and inter-community visits. After 18 months, a follow-up study will compare dengue history, serological evidence of recent dengue virus infection (via measurement of anti-dengue virus antibodies in saliva samples) and entomological indices between intervention and control sites.

**Discussion:**

Our hypothesis is that informed community mobilisation adds effectiveness in controlling dengue.

**Trial registration:**

ISRCTN27581154.

## Background

Recognition of dengue as a major health issue is increasing, especially in Asia and Central and South America [1]. In Nicaragua and Mexico, as in most dengue endemic countries, the national pesticide-dependent approaches have failed to curb the spread of this mosquito-borne disease. Several reviews have shown pace-spraying with pesticide to kill adult mosquitoes to be of little value [2]. Multiple serotypes of dengue virus continue to move northward through Latin America.

Failure to curb the dengue epidemic has led to resurgence of interest in community mobilisation for its vector control. A 2007 systematic review of community-based dengue control programmes, including two randomised controlled trials, found only weak evidence “that community-based dengue control programmes alone and in combination with other control activities can enhance the effectiveness of dengue control programmes” [3]. However, none of these earlier studies used clustered designs.

Several cluster trials have since shown an impact on vector densities. A team in Cuba published a trial of community mobilisation in 16 clusters compared with 16 controls, using vector breeding indices as the outcome [4]. In India, community-level provision of water container covers, clean-up campaigns, and dissemination of dengue information through schoolchildren also reduced vector density [5]. In Thailand, community volunteers reduced vector density [6]. No impact could be detected in a comparison of two communities in the Philippines [7].

A 2011 systematic review of 22 studies involving education messages for community-based dengue prevention concluded that these were effective in reducing entomological indices, but none measured dengue occurrence [8]. A recent systematic review of 14 studies of *Bacillus thuringiensis israelensis* found ample evidence that this reduced the number of *Aedes aegypti* breeding forms but only one study provided any evidence of impact on dengue risk; the study reported one dengue case in the intervention area and 15 cases in the control area [9].

The literature suggests that non-pesticide measures *should* prevent dengue, but there is very little direct evidence of this. A pesticide-free alternative has potential health, economic and environmental benefits.

### Feasibility study in Managua 2004–2008

#### Eligible participants

In Managua, Nicaragua, 10 intervention and 20 control sites (132 houses per site, 3,960 households and 3,300 children aged 3–9 years) piloted development of instruments and protocols for a pesticide-free intervention.

#### Willingness of participants to be randomised

There was no allocation concealment or randomisation to intervention and control. The feasibility study focussed on community intervention dynamics and development of impact assessment instruments.

#### Interventions

Baseline survey teams collected serological, entomological and behavioural evidence during biannual measurement cycles. Community volunteers used this evidence to promote dialogue on the issue of dengue prevention and to develop interventions in 10 sites. Interventions included household visits, active demonstration of mosquito eggs and larvae/pupae in the home, and use of simple tools and procedures for breeding site elimination.

#### Follow-up and response rates to questionnaires

Of 5596 households contacted in 2008, all allowed examination of vector reservoirs outside the building; all but 21 answered questions about recent illness in the household (99.7%).

#### The proposed outcome measures

Planned outcomes were serological IgG ELISA analysis of paired saliva samples to detect a decrease in the serological evidence of recent dengue virus infection in children aged 3–9 years; entomological indices; and household measures of social capital. Evaluation teams measured entomological, immunological and behavioural indicators each year. Covariates included use of temephos, type of water container, other containers near the dwelling, education of household head, involvement in other community matters, and number of people in the household.

#### Standard deviation of the outcome measure, to estimate sample size

In 2004–8, incidence of dengue virus infection in children over the two measurements ranged between 1.5% and 7.4% in the control communities; the reporting rate of dengue varied between 0.6% (1/166) and 5.3% (10/189); between 11% (20/183) and 50% (94/188) households were positive for *Aedes aegypti* larvae and/or pupae.

#### Adherence/compliance rates

The total 3,217 households with children aged 3–9 years yielded 2,992 saliva samples from these children (7% or 225 declined or were not present at the time of the visit). By the fourth year of the feasibility study (2007), twice as many households in the intervention sites (15.5%) as in the control sites (7.1%) said they participated directly in community dengue control exercises, and 50.7% in intervention and 39.5% in control sites reported having looked for larvae in their water stores over the last week.

#### Intraclass correlation coefficient (ICC)

Using an ANOVA framework for the 20 control communities in 2007, we estimated an ICC of 0.78 for the saliva samples of 3–9 year-old children and 0.396 for households positive for *Aedes aegypti* larvae and/or pupae.

#### Comment

As with many studies of this type [10], the feasibility study in Nicaragua was not randomised. We therefore did not evaluate the impact on dengue but estimated parameters needed to design the main study. The Nicaraguan study fine-tuned a dengue-specific intervention strategy called “socialising of evidence for participatory action” (SEPA). The study demonstrated feasibility of Camino Verde in vector control, with likely gains for the intervention communities [11–14]. The long history of community mobilisation in Nicaragua raises issues of replicability in other contexts. We therefore included southern Mexico in the main trial.

### Objectives of the Camino Verde trial

The research question is: Among residents of Managua and the State of Guerrero in Mexico, does evidence-based community mobilisation add effectiveness to the usual dengue control strategies to decrease the risk of dengue virus infection using a parallel group cluster RCT?

The Camino Verde hypothesis is that informed community mobilisation adds effectiveness in controlling dengue. The trial is concerned with the real-life effect of the intervention in the coastal region of Guerrero state and in Managua, rather than its effectiveness under ideal conditions [15, 16].

The overall objective is to reduce dengue risk through evidence-based knowledge translation. An explicit objective is to develop a sustainable community-oriented vector control approach that reduces the need for pesticides in water used in or around homes or areas that children frequent, or likely to be used with food or even as drinking water.

Specific objectives are (1) Determine entomological and serological status of the virus-vector-host ecosystem as well as knowledge, attitudes and behaviour underlying community-led interventions; (2) In partnership with intervention communities, translate this knowledge on dengue vector behaviour and immunology into household and community action against dengue; (3) Identify the impact of informed community mobilisation on dengue risk and its added effectiveness regarding other infections, social capital and longer term economic welfare.

## Methods/Design

### Design of the study

**Fig. 1:**
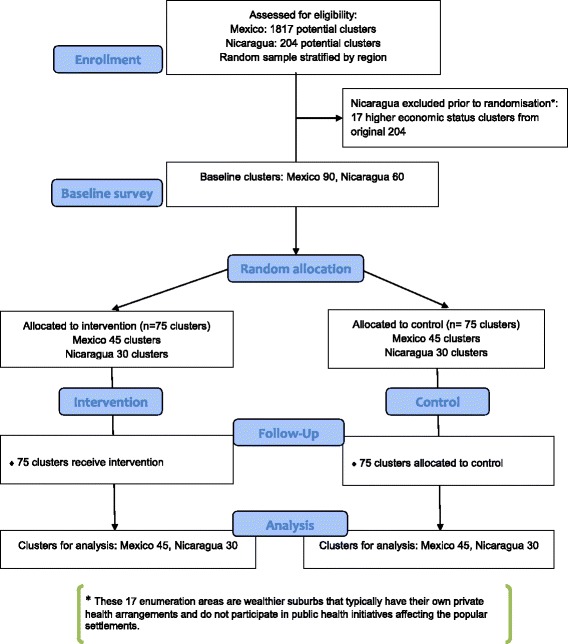
Camino Verde participant flow diagram

This parallel group pragmatic cluster randomised controlled trial will test informed community mobilisation in dengue control, measured by risk of dengue virus infection, occurrence of self-reported dengue cases, and vector density. The baseline will include a household questionnaire, entomological assessment and saliva samples from children aged 3–9 years. After the baseline survey, a sampling statistician not associated with the trial will randomise the 150 sites to intervention or control, stratified by area, entomological indices and serological evidence of recent dengue virus infection in children. Interventions will be based on evidence of *Aedes aegypti* infestation, explanation of the dengue vector life cycle, and information on how to prevent larvae and pupae from becoming adult mosquitoes. Different ways of transmitting this knowledge and acting upon it will be designed and implemented by the participating communities. The intervention will run for at least a full year. A multi-method impact survey will cover all 150 clusters with no in-cluster sampling. This will measure risk of dengue virus infection, disease reports, and entomological indices of the vector *Aedes aegypti*. Figure 1 is a flow-diagram of the trial.

### The setting

Nicaragua reports around 10,000 cases of dengue each year; all four serotypes circulate, with one serotype dominating in each season [17]. Between 2003 and 2007, registered dengue cases in Mexico increased 10-fold to 48,456. Guerrero now reports a high rate of dengue haemorrhagic fever (35/100,000), second in the country only to Quintana Roo, with all four serotypes well established. The Nicaraguan and Mexican government strategy for control of the *Aedes aegypti* mosquito rests on insertion of a packet of temephos (trade name Abate) in household water containers, known in both countries as “abatización”.

Participants are residents in a random sample of 150 clusters (100–120 households each) from the latest census in each country. In Mexico, a stratified last-stage-random sample generated 90 enumeration areas, 30 representing each of three regions (Costa Grande, Acapulco and Costa Chica regions of Guerrero state). In Nicaragua, 60 enumeration areas represent of the capital city, Managua, where approximately one-fourth of the Nicaraguan population resides.

### Interventions

“Socialising of evidence for participatory action” (SEPA) (http://www.ciet.org/en/method/sepa-communication/), was fine-tuned during the Managua feasibility study. Although there will be a high degree of local customising of the intervention, all sites will follow the same protocol to generate community-wide activities that vary widely in character and scope but all built around information about the life cycle of the mosquito and how to interrupt it:

1. Feedback of evidence from the baseline survey to community representatives in discussion groups to author the locally-defined interventions;
2. *Brigadistas* or volunteers from the communities will serve as organisers and as educators -- trained by facilitators from the research team, who will then be withdrawn from the communities -- conducting visits to schools and household (*visitas de acompañamiento*) to show the evidence of infestation in water receptacles;
3. Structural interventions will change the Aedes aegypti ecosystem in each intervention community;
4. Intercommunity visits and peer evaluations will share experiences between communities and strengthen the group dynamics and the collective preventive action.

In both intervention and control enumeration areas, interviewers from the baseline survey will return individual serology results and explanations of their interpretation to households where 3–9 year old children provided saliva samples. Other activities will depend on community preferences and, based on the feasibility study and discussions with pilot communities, could include neighbourhood clean-up campaigns, puppet theatre, basketball competitions, rap (reguetón) and other songs about dengue, parades, and poster competitions.

Community-to-community peer monitoring is an anticipated feature of the intervention, to be customised to suit the different conditions in the two countries. In Nicaragua, the four-year feasibility study has already provided a good idea of the change process so their emphasis may be more on the quantitative data. Both Mexican and Nicaraguan peer process evaluations will involve members of the research team from one cluster or region visiting another cluster or region and gathering both quantitative and qualitative information to provide project management and community leaders with actionable details about the intervention process, to inform any necessary mid-course corrections. In both countries, the inter-community visits and process evaluation will provide encouragement and training for intervention field staff and communities alike.

Quantitative information will include data on the last time a member of the cluster’s own research team visited the household and data on larval/pupal infestation as found by the monitoring team. Qualitative information will include recommendations from householders for improvement in the programme and responses to questions about the most significant change that community members have observed since the intervention began [18]. With the focus on the nature of the change, purposive sampling will yield a number of accounts to be processed [19].

### Outcomes

The main outcome measures are (i) risk of dengue virus infection as measured by a minimum of two-fold increase in anti-dengue virus IgG antibodies across paired saliva samples collected before and after the 2012 dengue season in children aged 3–9 years [20] (ii) dengue cases (any age) in the last year reported by the household respondent; and (iii) entomological indices of *Aedes aegypti* breeding sites, using the protocol developed in the Nicaragua feasibility study [21].

#### Serology samples

Interviewers who have not been part of the intervention will collect saliva samples from children (estimating ~140 households in each cluster, one child aged 3–9 years in every three households) in both intervention and control clusters at the beginning and at the end of the dengue season. During household visits, the interviewer will ask children to spit 0.5–2 ml of saliva into a plastic receptacle. Returning fieldworkers will transport samples to the CIET laboratories in Acapulco and the Ministry of Health National Virology Laboratory in Managua, divided into labelled aliquots and stored at −80 °C until processing. We will process the pre- and post-season samples side-by-side by capture ELISA and will consider an increase in IgG antibody units of greater than or equal to two-fold indicative of increased dengue virus infection risk. The analysis will be repeated for three-fold and four-fold changes.

The serology protocol requires incubation of polystyrene plates fixed with 100ul anti-human IgG antibodies (Sigma) incubated for 24 h, then incubated at 37 °C for one hour with 50 μl of undiluted saliva, followed by incubation for 1 h with 50 μl of antigen prepared from all four dengue serotypes prepared in-house in Nicaragua from infected mouse brain, with four washes in between each step. We will measure the colour change after adding 50 μl of anti-dengue virus immunoglobulin conjugated to horseradish peroxidase (prepared and titrated in Nicaragua) followed by 50 μl of TMB substrate (Sigma) at 450 nm in an ELISA reader. We will calculate antibody units as = [(OD sample - OD negative control)/(OD positive control - OD negative control)] × 100.

#### Breeding site assessment

Entomology evaluators who are unaware of the intervention status of sites will inspect indoor and outdoor containers, including natural habitats such as tree holes and leaf axils, that might harbour *Aedes aegypti* or *Aedes albopictus* immature forms (larvae or pupae). They will examine containers in dark or shaded areas using flash lights. Although recent research questions the value of *Aedes aegypti* larvae as a predictor of adult mosquito females in households [22–24], we include larval indices as both Mexican and Nicaraguan Ministries of Health rely on these for dengue mosquito surveillance [25]. Evaluators will examine all container types (tires, flower vases, water storage barrels, laundry wash basins, plastic tarps, puddles, and discarded containers (bottles, cans, drums, metal pots, plastic containers) [26]. They will collect all the larvae and pupae from each positive container using nets and pipettes, place them in labelled and capped plastic vials, and send them to the National Entomology Reference Laboratory in Nicaragua and CIET Entomology Laboratory in Mexico for identification. Double data entry with discrepancy verification will reduce keystroke errors before linking data with other study databases.

#### Entomology indices

These are a) House Index (HI), number of houses with one or more containers positive for immature *Aedes aegypti* divided by the number of houses sampled multiplied by 100; b) Container Index (CI) number of water-holding containers with immature *Aedes aegypti* divided by the number of water-holding containers inspected multiplied by 100; c) Breteau index (BI) number of water-holding containers with immature *Aedes aegypti* per every 100 households inspected; d) pupae per container or household inspected; and e) pupae per person (pu/per) number of pupae collected over the total number of inhabitants of the households inspected.

Secondary outcomes focus on the social capital gained from community engagement and the partial outcomes toward acceptance of the Camino Verde, including knowledge of and attitude to prevention, intention to change prevention behaviour, agency (collective and self-efficacy), discussion/socialisation, and prevention-related action. Additional secondary outcomes include purchase and use of pesticide, and reduced expenditure on health care as a consequence of dengue virus infection.

### Type of analysis

#### Data entry and security

The household responses to the questionnaires will be entered twice by independent operators who are ignorant of intervention status of the sites, with verification of discordant entries from the original questionnaires. A data manager ignorant of intervention status of the sites will check digitised data for logical errors. Questionnaires from intervention and control sites will be handled in exactly the same way, with all data technicians unaware of the intervention status of clusters. Site identities will be masked before analysis.

#### Principal analysis

With 150 clusters allocated evenly between intervention and control arms, the cluster analysis of primary outcomes will rest on a t-test, following an intention-to-treat principle (everyone included in each cluster, per allocation). The simultaneous evaluation in control and intervention sites will account for temporal effects. Serological status, reports of dengue illness, and vector indices lend themselves to analysis as continuous variables. Recent infection in children, adopting a cut-off of a two-fold increase of IgG units between samples, and pupae-positive households, are both dichotomized variables; the percent of households with serological evidence of recent infection in any child or with larvae/pupae-positive entomology serves as a continuous variable in each site. We will report outcomes as absolute event rates among intervention and control groups, with relative risk reduction (RRR) and 95% confidence intervals.

#### Secondary analysis

We will use individual-level data allowing for informative clustering in a non-linear mixed model with a robust variance and non-fixed odds-ratio [27] or generalised linear mixed model (GLMM) assuming cluster as a random effect [28]. We propose to examine average individual effects (for example, children with increased serological evidence of recent dengue virus infection, recent self-reported cases of dengue, or entomological parameters) while accounting for temephos exposure and water supply.

Sensitivity analysis will focus on gender-specific responses and exclusion of sites with prominent government pesticide intervention.

Planned subgroup analysis will focus on regional variations of the protective effect of Camino Verde, especially relating to population density (urban/rural) and the particular choices of intervention.

#### Missing data

All communities experience in-migration and out-migration. We will add new arrivals to the study but will not follow those leaving the household clusters. There is no reason to expect differential out-migration between intervention and control clusters. Self-selection (decision not to participate or not to answer certain questions) is a concern, given the structured engagement implied by the intervention. Those who opt not to respond may be less involved with dengue control – thus reducing the measured effect. We will analyse missing data using Amelia II [29] to impute values for missing data with an EM algorithm for the primary outcome (serological evidence of recent dengue virus infection). Estimates will reconcile data from ten imputed data sets using Rubin’s approach [30] in the R package Zelig [31].

#### Economic analysis

The sample size estimate for this trial uses relatively rare primary outcomes, so it will be adequately powered to consider economic implications of the intervention, which could be profound. Informed community mobilisation could reduce dengue rates directly but, by increasing social engagement, it could also increase the proportion of the population enabled to benefit from other prevention interventions, increasing their impact correspondingly. The dengue virus infection end-point (serology) is most relevant for economic analysis of the direct impact, although reduction of entomological indices also has resonance. By costing the intervention, it should be possible to document the costs of dengue cases avoided [32]. Documenting the real cost of preventing dengue will be less simple. A cost-benefit approach will be relevant, with benefits identified and qualified by communities themselves. Individuals who opt to reduce their own dengue risk probably do so based on perceived costs and benefits of prevention – we have called this the individual “cost benefit equation” [33]. This equation can lead to very different decisions from those imagined by dengue control programmes, especially in the context of abatización, which removes choice and changes the way people estimate their own value in the choices they make.

#### Implications for health services

The case to be made is that informed community mobilisation reduces dengue risks, without reliance on imported pesticide. A secondary case is that the costs compare favourably with the gains of doing this; pesticide-free prevention requires less treatment of cases, less work time lost, and less importation of expensive pesticides.

#### Software

CIETmap [34] is a hybrid vector-raster GIS software with seamless linkages between epidemiological analysis and mapping. The package provides a windows-like interface with the popular open-source statistical programming language R.

### Power calculation

Data from the feasibility study in Managua facilitated sample size estimation. Using the dengue serology results as the principal outcome, control communities in 2004–8 showed a range of 1.5% to 7.4% (k-statistic 0.328). Follow-up of 40 children in each of 150 clusters (75 clusters per arm) would detect a 33.3% reduction in incidence (4.5% to 3%) with 80% power at a significance level of 5%. These calculations assume no interaction effects and a two-sided test with cluster as unit of primary analysis in unmatched parallel groups. Calculations used a spreadsheet based on the trial simulator devised by Taylor and colleagues [35].

### Randomisation

#### Allocation concealment

Randomisation will be conducted centrally by a sampling statistician who is not engaged in the rest of the study. Computer-generated random numbers will determine intervention status in each stratum, defined by the baseline study. The intervention will begin promptly after randomisation with disclosure of intervention status at that time.

#### Blinding

The prevention efforts will be obvious to residents in the intervention sites, and some outcomes (particularly conscious knowledge) could be influenced by knowledge of intervention status. The main outcome indicators (reduction in serological evidence of recent dengue virus infection or entomological indices) would be less susceptible to this bias. To prevent potential bias, interviewers should ideally be unaware if they are interviewing in intervention or control clusters. Data managers will be unaware of intervention status of clusters.

#### Contamination

There may be some contamination of the control clusters: through people interacting between the clusters at the individual or household level; through schools (children from control clusters going to schools in the intervention clusters); and through local government or NGOs taking up the emerging evidence to guide interventions in control clusters. Perhaps a bigger concern, in urban areas, is that mosquitoes from neighbouring households influence rates in the intervention sites and that children may get bitten by infected mosquitoes outside of their intervention site [36, 37]. Both types of contamination will reduce the measured difference between control and intervention clusters. We cannot avoid this effect. We will document spread of the intervention beyond the sites in several regards: (i) mechanisms for generalising evidence, (ii) uptake at policy level and in the public discourse, (iii) new initiatives or changes in approach by agencies involved in dengue control, and (iv) impact in the control clusters as well as in the intervention clusters.

### Ethical considerations

#### Ethical review

The Institutional Review Board (IRB) at the University of California, Berkeley, and the CIET IRB reviewed the Nicaraguan arm. The Research Ethics Board of CIETcanada and the Ethics Committee of the Centro de Investigación de Enfermedades Tropicales at the *Universidad Autónoma de Guerrero* reviewed the Mexican arm.

1. Informed consent: Using a standard script, in the household survey, interviewers explain to respondents the nature of the survey, its voluntary character, and the anonymous quality of the process. They explain that respondents may decline to answer any questions they do not wish to, and may terminate the interview at any time. They then ask respondents for their consent to conduct the interview.
2. Samples: Separate informed consent is obtained from a parent or guardian for collecting the saliva samples with assurance that the results will be returned to them. To allow linkage of the repeat sample result with individual risk factors from the questionnaire responses, we use adhesive labels with ID codes fixed to the sample, to the completed questionnaire and, in the case of Nicaragua, to an administrative sheet that includes the date and time of the sample.
3. Ensuring confidentiality: Training of fieldworkers and data operators will emphasize responsibility for maintaining confidentiality of all information to which they have access during the work. We will report only grouped findings, in a way that does not allow identification of any individuals or individual communities. In the household survey, no names or identifiers will be recorded next to individual responses. Unique number labels will be used to link saliva specimens to questionnaire responses, without identifying the person by name. In individual follow-up for repeat samples, we will record the name of the child in order to match this from visit to visit and to be able to deliver results of the tests to parents or guardians. In focus groups, no names of focus group participants will be recorded, and reports of focus group findings will not identify individual communities.

4. Protection of emotional well-being: We do not expect the questions to awaken distressing memories. To the contrary, we expect the opportunity to engage in household and community protection to be uplifting and self-affirming.
5. Normative pressure within communities: Both the CIETinternational and CIETcanada ethical review boards raised questions regarding possible stigmatising of residents who failed to participate in community vector control activities. Both boards were satisfied by the responses they received from the field teams on this subject. We will prepare a full discussion of the ethical issues and our position regarding them and submit it for publication in a peer-reviewed journal.
6. Security of data: Digital records will be secure and accessible only to the lead epidemiologist. Original paper records will be securely transported, stored, retained and finally destroyed in accordance with CIET guidelines for security, storage and eventual destruction of paper records [38].
7. Promotion of equal opportunities: At least one half of field workers in the interview teams and the local action teams will be women. The field team leader in Mexico, Elizabeth Nava, is a member of the CIET academic council. Eva Harris is the nominated principal investigator of the Nicaragua team.

## Discussion

Current dengue control measures in Latin America rest on an organophosphate pesticide, temephos (Abate), inserted in the household water storage containers that are often breeding sites for *Aedes aegypti*, the main vector for dengue virus in the region. Many national governments have used temephos in household water stores. The WHO approval to do so [39, 40] cites the lack of evidence of toxicity.

Temephos is not licensed for human consumption in the USA [41]; the US Environmental Protection Agency says temephos “…has no food uses, is not likely to be found in drinking water, and is not used in or around homes or areas that children frequent” [42]. Studies not sponsored by the producers have documented animal toxicity [43, 44] and genotoxicity [45]. Resistance of the mosquito to this pesticide is already well documented and is a growing concern, given the variable coverage and consistency of many government-initiated programmes using temephos for dengue control [46–52]. We could not identify any community-based randomised controlled trials showing a protective effect of temephos against dengue virus infection.

Given the current government commitment to temephos, it will take a while to generate full participation of state and local authorities. Assuming the intervention is successful, there is an ethical obligation to implement it also in control communities. As this is unlikely within the 3-year time period of this project, other funds will be sought for that purpose.

There is also a strong community-building component integrated into the trial design; one of the goals is to measure the impact of the intervention on social capital. In each intervention cluster, the community action team will be trained in evidence-based communication as well as in ways of engaging with residents that are fully respectful of personal and community autonomy.
